# HIV prevention today: do we still need a vaccine? A community perspective

**DOI:** 10.1002/jia2.25806

**Published:** 2021-11-21

**Authors:** Maureen Luba, Ntando Yola, Rob Newells, Lilian Benjamin Mwakyosi

**Affiliations:** ^1^ Global Advocacy for HIV Prevention (AVAC) Lilongwe Malawi; ^2^ Advocates for Prevention of HIV in Africa (APHA) Cape Town South Africa; ^3^ Black AIDS Institute Atlanta Georgia USA; ^4^ DARE Organization Dar es Salaam Tanzania

**Keywords:** HIV vaccine, vaccine efficacy, community engagement

Despite tremendous advances made in the global HIV response, overall progress in HIV prevention efforts remains too slow to reach the 2030 targets [1]. In 2019 alone, 1.7 million people acquired HIV, three times more than the UNAIDS 2020 target [2]. About 62% of the new infections were among key populations, these are men who have sex with men, sex workers, transgender people and people who inject drugs, and their sexual partners, and one in four new infections in sub‐Saharan Africa are among adolescent girls and young women [2]. Ending HIV as a global health threat will require rapid scale up to near universal coverage of high impact HIV prevention and treatment interventions [3].

Thanks to research and development, more HIV prevention tools are now available or in the research pipeline. However, the current tools remain inaccessible to the majority of those who need them either due to access challenges; their cost, availability, structural barriers to access as well as challenges with their use; adherence, and personal choice and preference. Therefore, it is believed that achieving universal coverage of high impact HIV prevention tools will still require developing additional tools, including a safe and effective vaccine.

Since the approval of the first oral HIV prevention pill, it has become commonplace to hear declarations that we now have the tools we need to end HIV. These statements discount access, uptake and preference challenges. In the last decade, we have added oral, vaginal ring and injectable PrEP and long‐acting treatment to the existing toolbox of highly effective HIV prevention strategies, but we still need a highly effective vaccine to end the global pandemic for good.

Vaccines are liberating – Most vaccines offer long‐term protection against diseases [4]. The measles vaccine provides lifelong protection, and others, including vaccines for tetanus, polio and hepatitis B, provide protection for 10, 20 or more years. People who have been vaccinated do not have to think about protecting themselves from the diseases against which they have been vaccinated. Adherence and the need for regular engagement with the healthcare system becomes less of an issue.

Vaccines are easy – Healthy young people and those who do not see themselves at risk for HIV do not prioritize protecting themselves against HIV, especially if doing so requires payment and accessing unfriendly healthcare systems. An effective safe and durable vaccine would go far in meeting the needs of many different populations.

“*Even in the absence of an effective HIV vaccine, which would be the final nail in the coffin of the pandemic, we have the tools to end the HIV/AIDS epidemic in the United States and globally*”. *–Dr. Anthony Fauci [* *5* *]*.

We agree with Dr. Fauci – “We still need an effective HIV vaccine to bring an end to the HIV pandemic”. Science has shown us that developing an HIV vaccine has been quite challenging because the HIV mutates rapidly and has its own unique ways of evading the immune system. It has been over three decades since the first human trial of an HIV vaccine candidate was conducted in the United States. To date, no licensed vaccine exists, but multiple clinical trials are underway to find an effective HIV vaccine.

The story is different for COVID‐19. Decades of HIV vaccine research and advocacy made COVID vaccines possible. In less than a year, the global COVID 19 response has produced the fastest, best coordinated and funded vaccine development effort in history, leading to the discovery of multiple highly effective vaccines in record time. The global partnerships and the political and financial commitment demonstrated in the development of COVID‐19 vaccines and therapeutics should be replicated in the HIV vaccine research and development space.

Besides the critical RV144 trial, there have been a number of efficacy trials which have been stopped for futility, including the most recent Uhambo (HVTN 702 trial) [6]. Uhambo was a phase 2b study which began in 2016 to evaluate the preventive vaccine efficacy, safety and tolerability of ALVAC‐HIV (vCP2438) + Bivalent Subtype C gp120/MF59 in HIV‐seronegative South African adults over 24 months and potentially up to 36 months from enrolment [7]. This trial was based on the positive results of RV144 and its cancellation has been a blow to the HIV community. There are, however, other opportunities in the pipeline.

After RV144 reported estimated cumulative efficacy of 60.5% in the 12‐months after initial vaccination, Dr. Robert Gallo was quoted as saying “You could make the argument that a vaccine could be licensed if it reaches 60%” [8]. The current approved COVID‐19 vaccines have set the efficacy bar very high. And now the question is, will communities accept an HIV prevention vaccine with less than 60% efficacy? Community preparedness for HIV vaccine trial results will be key. There will be a need for strong collaboration between researchers and community advocates in the provision of HIV vaccine research literacy and preparing the communities for whatever results we might end up getting.

The dosing and the timing between doses is another important key consideration. The HIV vaccine development community should draw lessons about acceptability, access and user preference on dosing and timing from COVID 19 vaccine rollout programs. These questions should also be embedded in the design of current and future HIV vaccine trials. Preparing for the rollout of long‐acting injectable PrEP products presents potential opportunities to learn about what an HIV vaccine rollout and preferences could look like.

COVID 19 has also exposed alarming rates of vaccine hesitancy, which is likely going to be a major challenge for HIV vaccine development and rollout, necessitating the need for robust community engagement during ideation and implementation of clinical trials as well as vaccine rollout to clear doubts, myths and misinformation about vaccine science.

HIV remains a global pandemic that kills more than 700,000 every year. Finding an effective HIV vaccine is not an option. It is a must. Development of a vaccine to prevent HIV would relax requirements for frequent visits to HIV specialists, pharmacy and laboratory services as well as reduce the associated out‐of‐pocket healthcare costs.

## COMPETING INTERESTS

All authors confirm that they have no competing interests.

## AUTHORS’ CONTRIBUTIONS

ML, NY, RN and LBM contributed to the conceptualization and design of the viewpoint and wrote the manuscript.

## FUNDING

None.
